# Document-Engineering Methodology in Health Care: An Innovative Behavioral Science–Based Approach to Improve Patient Empowerment

**DOI:** 10.2196/19196

**Published:** 2020-09-28

**Authors:** Bernd Pohlmann-Eden, Silke C Eden

**Affiliations:** 1 Department of Pharmacology & Toxicology University of Toronto Toronto, ON Canada; 2 Problem-Based Online Health Consultancy Toronto, ON Canada

**Keywords:** document design, 1-pager, empowerment, patient engagement, cognitive science, health care, cross-industry thinking, malpractice in health care, written information

## Abstract

Engaging patients in their treatment and making them experts of their condition has been identified as a high priority across many medical disciplines. Patient empowerment claims to improve compliance, patient safety, and disease outcome. Patient empowerment may help the patient in shared decision making and in becoming an informed partner of the health care professional. We consider patient empowerment to be in jeopardy if written medical information for patients is too complex and confusing. We introduce document-engineering methodology (DEM) as a new tool for the health care industry. DEM tries to implement principles of cognitive science and neuroscience-based concepts of reading and comprehension. It follows the most recent document design techniques. DEM has been used in the aviation, mining, and oil industries. In these very industries, DEM was integrated to improve user performance, prevent harm, and increase safety. We postulate that DEM, applied to written documents in health care, will help patients to quickly navigate through complex written information and thereby enable them to better comprehend the essence of the medical information. DEM aims to empower the patient and help start an informed conversation with their health care professional. The ultimate goals of DEM are to increase adherence and compliance, leading to improved outcomes. Our approach is innovative, as we apply our learning from other industries to health care; we call this cross-industry innovation. In this manuscript, we provide illustrative examples of DEM in three frequent clinical scenarios: (1) explaining a complex diagnosis for the first time, (2) understanding medical leaflet information, and (3) exploring cannabis-based medicine. There is an urgent need to test DEM in larger clinical cohorts and for careful proof-of-concept studies, regarding patient and stakeholder engagement, to be conducted.

## Setting the Scene

The only thing more expensive than education is ignorance.

Benjamin Franklin

Fortunately, modern medicine in the second millennium provides people in need of health care a constantly growing range of options, both in the diagnostic field and in the treatment field. Leading the way are the vast resources of medical information available on the web. Paradoxically, the described scenario can be overwhelming for the individual patient who finds it hard to navigate an increasingly complex health care system and make the right choices. In this manuscript, we postulate that there is a real need for well-designed and easy-to-understand written medical information to get patients engaged, informed, and ultimately empowered to positively impact their own disease outcomes.

Active engagement of patients and patient-centered care have been recognized for decades as priorities [1,2]; it has been suggested more specifically to enlist patients and families as allies in designing, implementing, and evaluating health care systems [1]. These concepts, driven by the vision to make the patient the expert, resulted in shared decision making, improved compliance, and improved adherence to medication [3]. Encouraging patient participation and self-management helped patients to gain control over their medical conditions and ultimately feel empowered [4,5]. How best to engage patients, doctors, and other stakeholders in designing comparative effectiveness studies has become an extensive field of research [6-8]. There is an ongoing need to investigate the dividends of engaged research and how to evaluate these effects [9].

**Figure 1:**
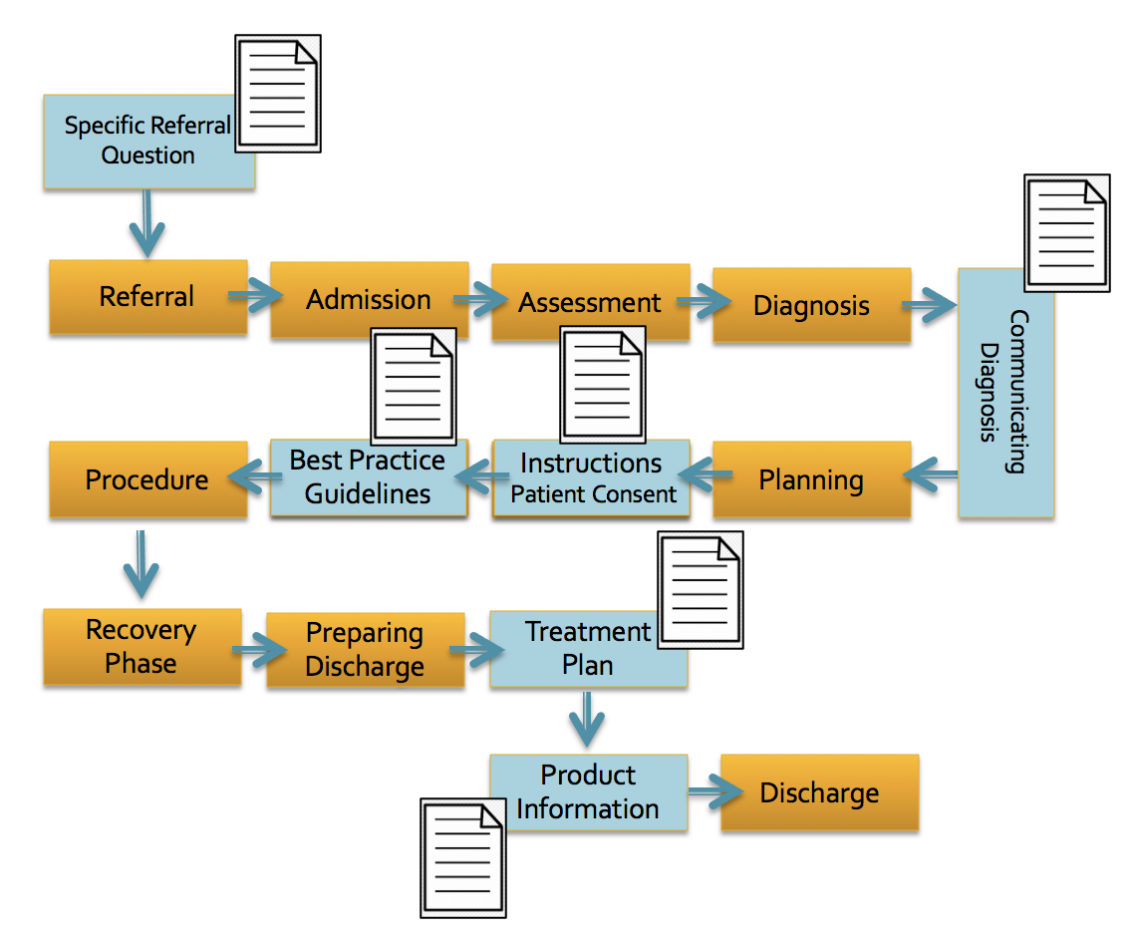
There are multiple steps in the successful delivery of health care with critical phases, where clearly written and easy-to-understand communication documents are key.

Despite all these efforts, medical mistakes and malpractice still occur on a large scale. In North America, the number of people dying in hospitals as a result of malpractice and adverse drug events exceeds the number of deaths as a result of car accidents [10]. In a seminal paper almost 20 years ago—*No Toyotas in health care: Why medical care has not evolved to meet patients' needs—*the missing “business case of quality” in health care was criticized [11]. Meanwhile, many health care organizations adopted the Toyota Production System as the performance improvement approach, often called the LEAN health care management system [12]. The LEAN improvement process focuses on defining value from the patient point of view, mapping value streams, and eliminating waste in an attempt to create continuous flow [12]. These attempts are in line with the extensive quality improvement movement, which aims for better patient and population outcomes, better professional development, and better system performance [13]. Surprisingly, the scope of insufficiently written documents for malpractice in health care has never been systematically assessed in an epidemiological study. This finding is an interim result of an ongoing, not-yet-published, PhD research project at the University of Heidelberg, Germany, under supervision of the main author (BP). This is surprising, as written documents are used routinely at multiple intersections of an individually complex health care delivery process. These intersections include referral letters, information brochures about diseases, product information, consent forms, procedure guidelines, and treatment protocols (see Figure 1).

This is also in contrast to the fact that health literacy—the ability to read, write, and understand—has been recognized as an important milestone of the empowerment learning process for patients [14]. Health literacy allows the patients to perform knowledge-based literacy tasks in order to acquire, understand, and use health information for making their own health-related decisions. It has been postulated that these skills—applied in various environments, such as a home, community, or health clinic setting—will help the informed patient to prevent medical mistakes and increase their safety [15].

Lack of health literacy with subsequent misinterpretation of written material is still a current concern. In the European Health Literacy Survey, 1 in 2 (47%) out of 8000 participants in eight different European countries had limited (ie, insufficient or problematic) health literacy [16]. Several studies confirmed that lack of health literacy has significant impact on safety, specifically on desired patient health outcomes. These include higher rates of medication errors as a result of misinterpretations of prescription drug label instructions [17], reduced patient recollection and understanding of informed consent [18], decreased cancer screening and immunization rates, and, finally, more emergency department use [19]. Furthermore, a very recent systematic review evaluated the readability of online health information in the United States and Canada: based on 3743 references, 157 cross-sectional studies, and 13 different scales, the mean readability grade level was by far too difficult to comprehend for the targeted audience. It ranged from grades 10 to 15, while a grade 6 reading level for the general public is recommended [20].

In the following section of this paper, we will introduce document-engineering methodology (DEM) for designing medical information. The idea of DEM comes from industries such as aviation and oil, which proposed that DEM will help users to prevent errors, measurably reduce risk for injuries, and, overall, increase safety by designing an easy-to-read document [21]. In an innovative approach, we introduce DEM for the first time to the medical field.

## Document-Engineering Methodology: A Cognitive Science–Based Approach?

It has been well known for more than 100 years that the brain is not perfect at all; it naturally produces errors while receiving, selecting, and processing information. We will provide two famous examples from cognitive neuropsychology and behavioral science.

In 1907, the Hungarian neurologist and psychiatrist Bálint wrote, “It is a well-known phenomenon that we do not notice anything happening in our surroundings while being absorbed in the inspection of something; focusing our attention on a certain object may happen to such an extent that we cannot perceive other objects placed in the peripheral parts of our visual field, although the light rays they emit arrive completely at the visual sphere of the cerebral cortex” [22].

The natural limitation of the brain to process and identify all visual information at the same time was further supported by the behavioral experiment of Simons and Chabris [23]. In their seminal paper, the authors describe an experiment in which a dancing gorilla was entirely missed on a video by observers when they were told to strictly focus on ball contacts of two teams of basketball players playing in front of the dancing gorilla. This phenomenon was subsequently called “inattentional blindness.”

Document design as a new research field integrated these basic insights of the brain processing visual information and added several other components. Karen Schriver, an early scientist in technical writing, pioneered this approach. Her groundbreaking, extensive research is summarized in the comprehensive textbook *Dynamics in Document Design: Creating Text for Readers* [24]. Her insights about writing, reading, and visualizing documents defined the art of document design. The author emphasizes the importance of typography and space to improve readability and communication. Well-known principles of Gestalt psychology (ie, closure, symmetry, asymmetry, proximity, similarity, continuity, grouping, hierarchy, and balance) are implemented in the framework of document design [24].

Document design, with the main question on how we process and *read* written information, has been influenced by a multidisciplinary field of research. It spans over four decades and ranges from the classic psychological *theory of reading* by Just and Carpenter [25] to studying neuronal networks and circuits via advanced magnetic resonance imaging techniques while reading. The focus of this research was on visualization of subtle sequential processing steps within the brain while reading [26,27]. Other studies addressed the role of eye tracking for scanning and skimming written information, an issue that gets even more important in a fast-paced modern world using short messages for information dissemination on smartphones and other portable devices [28].

More recent research focuses on the *user* perspective in industry and how the user processes and *reads* procedural instructions [29]. The author suggests that the user *consults* a document in an interactive way rather than reading it in a linear manner [29]. Document design factors based on cognitive neuropsychology are introduced to allow reading with understanding, action planning, carrying out specific actions, and executive control activities [29]. These document design characteristics include a chronological or modular organization of the text, clear and precise headings, and using textual instructions where the word order strictly corresponds with the required action, question, or task to fulfil [29]. Design rules and design models based on cognitive and perceptual science have been proposed to further support engineering methods for interactive system design [30].

**Figure 2:**
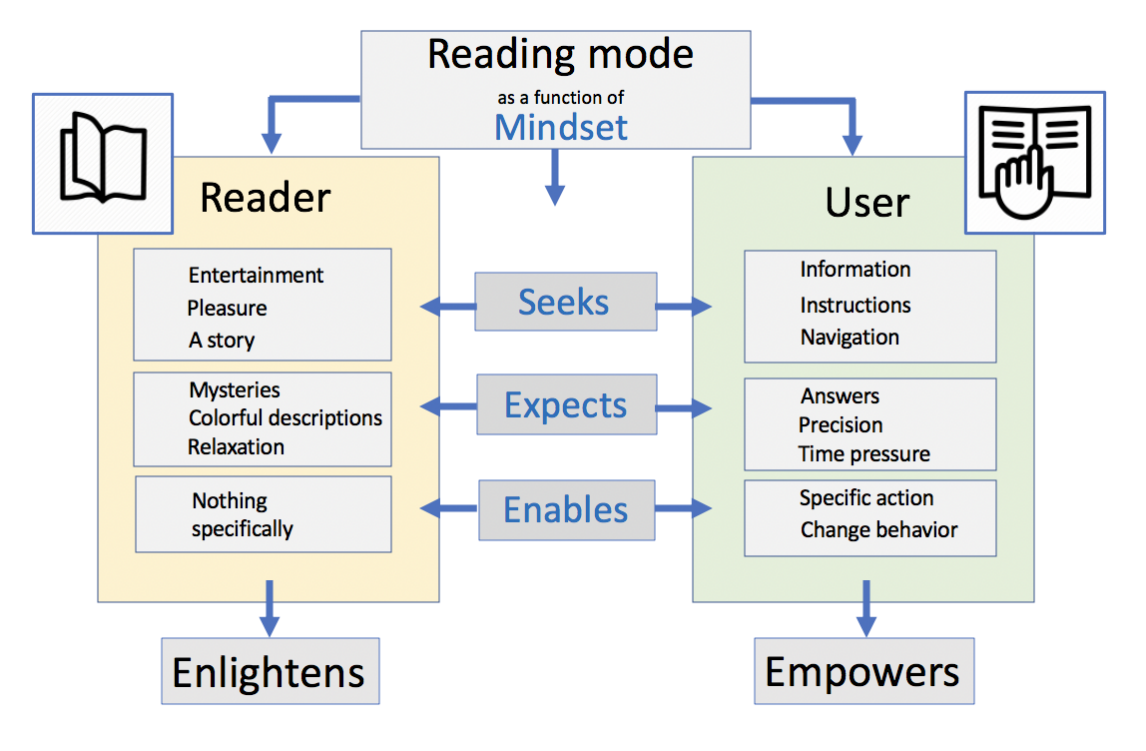
Two mindsets of processing and perceiving written information: readers reading versus users reading [31].

These approaches are in line with our recently proposed model [31] that *readers* (of books) and *users* (of written information) have different mindsets (see Figure 2 [31]). While the mindset of readers is driven by curiosity (ie, seeks reading for entertainment), users want to have immediate answers to their questions, often with a sense of urgency. Users need to be able to quickly navigate written information and need to be enabled to perform a specific action [31]. Recognizing the different mindsets between a reader and a user has enormous implications for designing a document.

The original term *document engineering* comes from the software and hardware computer industry [32]. In its strictest sense, it is a document-centric synthesis of complementary ideas from information and systems analysis, electronic publishing, business process analysis, and business informatics. It attempts to unify these different analysis and modeling perspectives and helps to specify, design, and implement documents and the processes that create and consume them [32].

The way we will use the term *document engineering* is quite different from the original description. We define DEM as an innovative subspecialty methodology of document design–implementing principles of cognitive science and neuroscience. The *engineering* part in our approach to DEM refers to our process of *putting parts together* of the outlined frameworks required to process written information in the most effective way [24-29]. Applying this current scientific knowledge, we hypothesize that DEM will enable the user, in our case the patient, to easily read and understand written information and to perform actions and tasks quickly, safely, and efficiently.

Several industries outside of health care have used DEM in order to improve user performance, prevent harm, and increase safety. Proof-of-concept research studies are unfortunately missing. The biggest lessons learned come from the aviation industry, where safety is the number one priority and difficult-to-read, user-unfriendly information has repeatedly caused fatal and avoidable incidents [33].

Corporate psychology in the oil and gas industry has also applied this behavioral science–based methodology to help the brain *navigate* more easily through complex document-based information, such as procedural instructions. However, the statement “The user is enabled to take the right action fast and efficiently with measurably reduced risk of harm, hereby increasing safety” [21] still needs reconfirmation through practical research-based trials.

## DEM-1-Pager to Ease Communication in Health Care

We suggest use of the DEM in health care. It is an opportunity to further establish the methodology and to test its added value in controlled trials. We provide three illustrative examples for potential use of a *DEM-1-pager*. In all three proposed examples, we produced an easy-to-read, single-page document, following DEM. The two authors of this paper pioneered and introduced the concept of *DEM-1-pagers* to health care only recently [31]. We use this as our *first* example in this manuscript.

As our target group, we chose people with a complex brain disease called psychogenic nonepileptic seizures (PNES). We sensed the suffering and the confusion of the people affected by PNES as we talked with them. They expressed, in particular, their frustration regarding insufficiently easy-to-understand learning material about their condition when communicating with their health care providers. People with PNES struggle with several challenges [34]. They face the overwhelming complexity of their disorder, they do not understand the underlying causes and prognosis, they recognize the lack of education around all stakeholders, they experience lots of obstacles and barriers in the health care system, and, most importantly, they are ill- informed right from start of their diagnosis of PNES [31,34].

Our way out of this dilemma was to produce a new communication tool in close collaboration with PNES patients: a *DEM-1-pager*. Our *DEM-1-pager* is content engineered for users—it is not written for readers.

We used a user-friendly, promise-question-answer (PQA) format as introduced in the oil and mining industry through corporate psychologists [21]; BP, one of the authors, is certified for this methodology. The PQA table is a basic framework with a heading and two columns; it consists of a *promise* presenting as the heading of the document (ie, the overriding topic the reader can expect). Organized on the left side of the document in a separate column are the most relevant *questions*. On the right side of the document are the *answers* strictly addressing the questions in simple terms.

We controlled for easy comprehension and readability by using a low Flesch-Kincaid reading level of seven [35]. The Flesch-Kincaid grade level is calculated by using a statistical program and the Flesch-Kincaid Grade Level Formula. The complex formula considers the number of words and syllables within a sentence. It measures the simplicity of writing and is widely used by teachers, librarians, educators, and others to assess the readability level of written text. We further embedded document design techniques from behavioral and Gestalt psychology [24,30]. The most important ones were limiting the questions to list to a maximum of seven items [36], implementing *cognitive linking* (ie, questions and answers containing similar wording) [29], and using behavioral enforcers [29]. We are aware that the “magic number of seven” has initiated a controversial discussion among neuropsychologists; it is also an excellent illustration for a frequent dilemma in cognitive science–based experimental findings. A rather low amount of research has followed on the numerical limit of capacity in working memory [37,38].

The outlined design techniques will enable the patients to navigate fast and efficiently through this document and quickly find answers to their pressing questions. Our tool provides the patient with the most important, essential information about PNES, including the relevant obstacles from the health care system. Our *DEM-1-pager* is not meant to replace available comprehensive and often time-consuming information either published on paper or online [39]; rather, it is meant to be complementary to these valuable resources. Ideally, it can be used in the initial communication between PNES patients and health care professionals.

**Figure 3:**
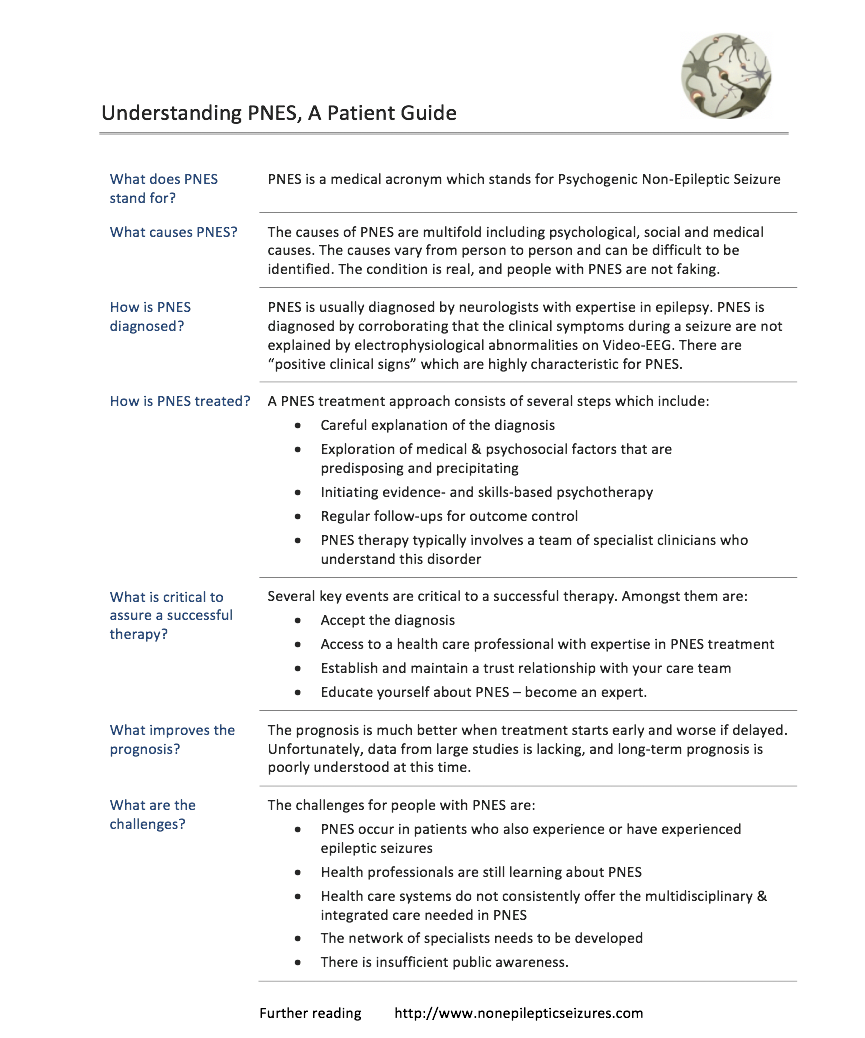
Document-engineering methodology (DEM)-1-pager for psychogenic nonepileptic seizures (PNES) (version 4); a tool for early communication of PNES created in a design-thinking process with patient engagement [31].

We engaged a group of PNES patients and cocreated with them the *DEM-1-pager* using a design-thinking process with many iterations [31]. We subsequently tested our *DEM-1-pager* in a small focus group of PNES patients; it was found to be beneficial in several domains. It also empowered patients to make their own decisions [31]. Figure 3 [31] shows the final version of a *DEM-1-pager* for PNES. The result is a poignant *DEM-1-pager* without overwhelming and confusing information.

Textbox 1 lists a range of other, randomly chosen, frequently occurring, complex medical conditions in which a *DEM-1-pager* can be helpful and contribute to early patient engagement.

**Examples of complex diseases in which a document-engineering methodology (DEM)-1-pager of information could be useful.**

- Psychogenic nonepileptic seizures
- Autism spectrum disorder
- Bipolar disorder
- Posttraumatic stress disorder
- Attention deficit hyperactivity disorder
- Diabetes mellitus
- Colon cancer
- Parkinson disease
- Fibromyalgia
- Chronic fatigue syndrome
- Alzheimer disease
- Many more diseases

As a *second* example, we chose patient information leaflets. Information leaflets are purposefully exhaustive and detailed in order to meet all medico-legal requirements. Patients often feel overwhelmed with the extent of written medical information, find it useless, and even tend to throw it away [40]. Patient information leaflets often are extremely wordy and not well designed and patients find it hard to navigate them. The leaflets almost never have a grade 6 readability level as a basic requirement. They often do not meet patients' needs and appear ineffective [41]. Patients cannot find the information they seek or may be confronted with nonessential material, affecting patients' perceptions of the leaflets and willingness to read them [42]. Applying DEM principles to information leaflets will hopefully reduce redundant words, improve format and design, and take health literacy (ie, grade 6 readability) into account.

As stated earlier, we do not suggest replacing *patient information leaflets—*we do see the necessity to present medico-legal information in the most complete and comprehensive way. However, we believe a complementary, easy-to-read *DEM-1-pager* will enhance the willingness of the patient to consider their suggested medication, for example.

**Figure 4:**
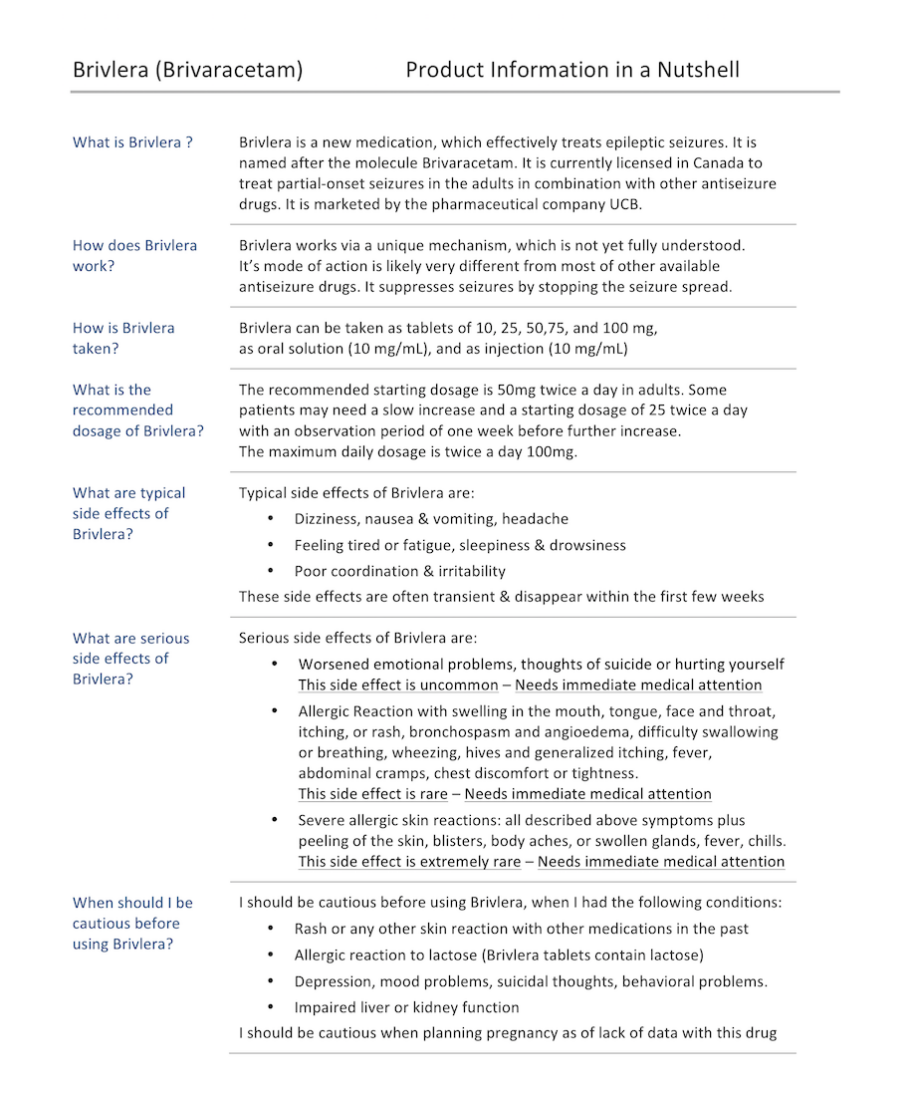
User-friendly, document-engineered methodology (DEM)-1-pager for the antiepileptic drug brivaracetam.

We provide an illustration of this approach. The lead author of this paper (BP) is a seizure expert and subject matter expert. He applied DEM to a comprehensive, 18-page, official US Food and Drug Administration (FDA) patient information leaflet for brivaracetam, a newly licensed medication for seizure control [43]. The result is a *DEM-1-pager* (see Figure 4) that contains all essential information. The *DEM-1-pager* can help to start an initial communication about brivaracetam. Readability of a document encourages the patient to be compliant and become an informed partner. The 18-page FDA information leaflet is a critical complementary resource at any time.

The *third* example shows a *DEM-1-pager* that we purposely developed for an extremely controversial uncharted territory: the new field of medical cannabis-based medicine (CBM). Though cannabis has been employed medicinally for more than two millennia, its recent legal prohibition, biochemical complexity and variability, quality control issues, previous dearth of appropriately powered randomized controlled trials, and lack of pertinent education have conspired to leave clinicians in the dark as to how to advise patients pursuing such treatment [44]. The use of CBM is still stigmatized, and health care providers are often reluctant to prescribe it. This is in contrast with the promising potential of CBM for multiple disorders and established clinical indications, such as epilepsy and pain [45].

The main author of this paper (BP) and other subject matter experts identified CBM as an ideal application for the use of a *DEM-1-pager*. Patients who seek treatment for chronic pain, one of the most accepted and evidence-based indications for CBM, want basic information about how CBM works. They are often desperate and seek knowledge through dialogue with their health care providers. These patients often encounter difficulties in finding answers to their most burning questions. They are confused and need navigation. Patients want to know how CBM might help them, information about side effects, how CBM can be consumed, how CBM is prescribed, which challenges they may face in the health care system, and so on.

**Figure 5:**
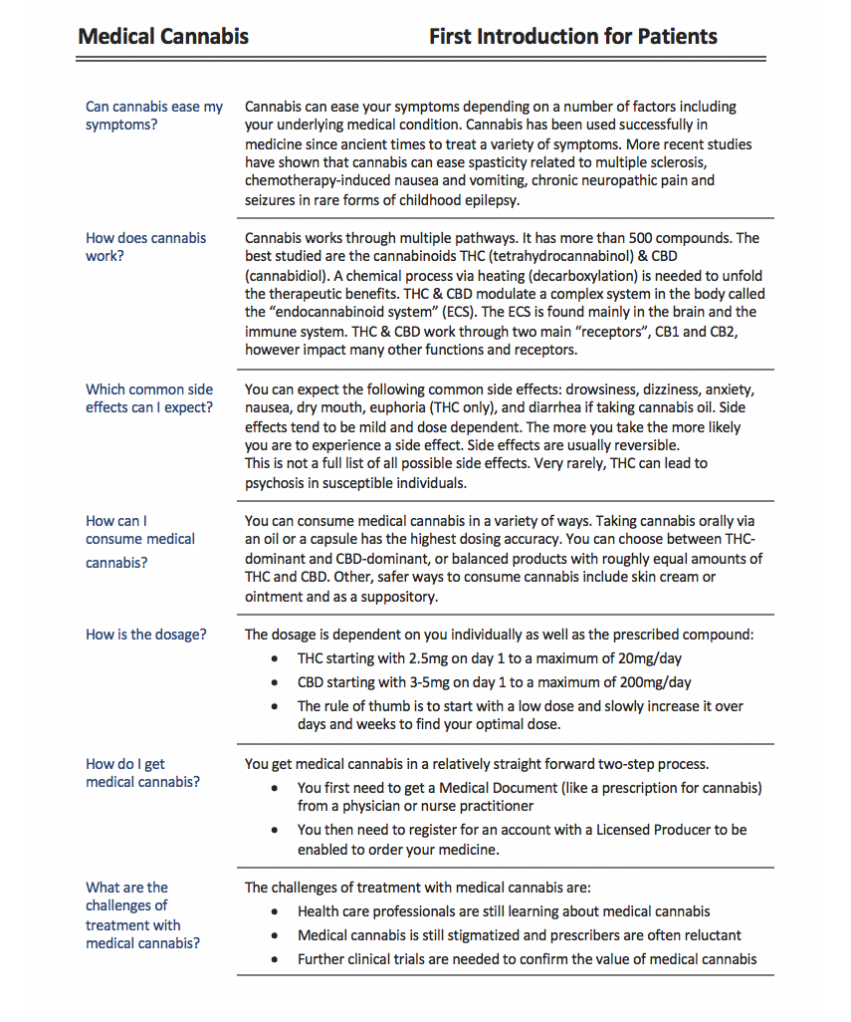
Proposed document-engineering methodology (DEM)-1-pager for patients interested in medical cannabis.

Figure 5 shows a proposal of an easy-to-read *DEM-1-pager* addressing this patient problem. This document was created in a design-thinking process together with subject matter experts. It aims to help patients to easily find answers for their most relevant above-mentioned questions. This *DEM-1-pager* is a perfect start for a first dialogue between health care providers and patients on the topic of CBM. It is not meant to replace other valuable comprehensive resources.

There are several limitations of the three provided examples of *DEM-1-pagers*. Only the first example, dealing with psychogenic nonepileptic patients [31], actively involved patients and health care professionals. This allowed a critical design-thinking process with reiterative feedback from users. The second and third examples lacked this process and still have to undergo testing in a focus group or in specific target groups. Some of the written content could certainly be replaced by colorful images to ease reading and understanding [29]. Active involvement of patients in designing these images is another intriguing opportunity for further templates.

We also see potential risks in using the presented *DEM-1-pagers*. They will always be simplifications of complex medical information. This goes along with the risk of likely not covering all individually highly relevant aspects. The patient may not seek out the more detailed complementary information, even when encouraged. This could harm the patient. It is, therefore, critical that the health care professional always explain the limitations of this tool to the patient.

## Conclusions

Our paper encourages the consideration of *DEM-1-pagers* in several health care delivery environments where written medical information is relevant, complex, and widely used (see Figure 1), such as referral documents, consent forms, and instructions for treatment procedures, to name a few.

We anticipate that *DEM-1-pagers* will help health care professionals to initiate and strengthen the dialogue between the health care professional and the patient, helping to build trust. This can lead to empowerment on both ends. A *DEM-1-pager* is conceptualized to be a first step to explain essential information, followed by a more sophisticated and detailed discussion on the subject later on. We hypothesize that *DEM-1-pagers* will help to improve patient guidance, empower the patient, and, ultimately, contribute to better outcomes.

We foresee a wide range of potential applications in the health care industry. We are fully aware of the limitations of our pilot data. Strong evidence is still lacking. Larger test studies will be needed to further validate *DEM-1-pagers* in various clinical scenarios. We, therefore, fully agree with a recent research paper mapping hypothesized impacts to suggested and assessed measures of patient, public, and stakeholder engagement. Their careful assessment confirmed lack of evidence underlying much of the impetus behind the practice of patient and stakeholder engagement in research, based on analyzing peer-reviewed literature using PubMed and PsycINFO databases from January 2005 to May 2013 [9].

We are also aware that we could not address all aspects of the impact of DEM in health care. It is, for example, beyond the scope of this paper to outline the health-economic and medico-legal aspects of patient and user empowerment by means of *DEM-1-pager*-designed documents. We also did not address the health-related preventive nature of well-written information; for example, poorly written child safety seat installation instructions have been found to be potentially harmful [46].

The main purpose of our paper is to encourage health care professionals to think in new ways about written medical documents for patients. The lessons from other industries about the usability of documents are intriguing. Cross-industry thinking carries a treasure of opportunities and will also facilitate breakthrough product innovation [47]. Safety is at stake if we do not open up to accept well-recognized and researched performance measures in these very industries. Health care is certainly still far behind in producing well-designed and user-friendly documents. DEM is a first step in this new uncharted territory.

## Abbreviations

CBM: cannabis-based medicine
DEM: document-engineering methodology
FDA: Food and Drug Administration
PNES: psychogenic nonepileptic seizures
PQA: promise-question-answer
